# Metro passengers’ route choice model and its application considering perceived transfer threshold

**DOI:** 10.1371/journal.pone.0185349

**Published:** 2017-09-28

**Authors:** Fanglei Jin, Enjian Yao, Yongsheng Zhang, Shasha Liu

**Affiliations:** 1 MOE Key Laboratory for Urban Transportation Complex Systems Theory and Technology, Beijing Jiaotong University, Beijing, China; 2 School of Traffic and Transportation, Beijing Jiaotong University, Beijing, China; 3 State Key Laboratory of Rail Traffic Control and Safety, Beijing Jiaotong University, Beijing, China; Beihang University, CHINA

## Abstract

With the rapid development of the Metro network in China, the greatly increased route alternatives make passengers’ route choice behavior and passenger flow assignment more complicated, which presents challenges to the operation management. In this paper, a path sized logit model is adopted to analyze passengers’ route choice preferences considering such parameters as in-vehicle time, number of transfers, and transfer time. Moreover, the “perceived transfer threshold” is defined and included in the utility function to reflect the penalty difference caused by transfer time on passengers’ perceived utility under various numbers of transfers. Next, based on the revealed preference data collected in the Guangzhou Metro, the proposed model is calibrated. The appropriate perceived transfer threshold value and the route choice preferences are analyzed. Finally, the model is applied to a personalized route planning case to demonstrate the engineering practicability of route choice behavior analysis. The results show that the introduction of the perceived transfer threshold is helpful to improve the model’s explanatory abilities. In addition, personalized route planning based on route choice preferences can meet passengers’ diversified travel demands.

## Introduction

The Metro is widely regarded as an energy-efficient transport mode to undertake the task of enormous resident trips in densely populated urban areas, which is capable of improving the urban traffic effectively. Prioritizing public transportation systems, particularly the Metro, is considered one of the most effective strategies to reduce car dependency, mitigate traffic congestion, and alleviate air pollution. [1]

With the rapid development of the Metro in China, particularly in certain megacities, such as Beijing, Shanghai, and Guangzhou, the network keeps expanding rapidly in both size and complexity. [2] This situation leads to the diversity of route choice and the variation on the spatial and temporal distribution regularities of passenger flow. [3] Moreover, given that huge passenger flows generated throughout the day place great strain on the Metro network and bring challenges to operation management, explaining the route choice decision-making process of passengers is particularly important for daily operation management. The route choice behavior analysis results can be applied to various aspects, such as passenger flow assignment, transfer volume forecasting, personalized route planning, and fare clearing, which can be further used in advanced passenger information services and are essential for the operation department to adjust operation plans and management.

Based on prior studies, analysis on passengers’ route choice preference has been emphasized in recent decades. There are four categories of explanatory variables that influence passengers route choice behavior, including the level of service (LOS) variables, the socioeconomic and demographic characteristics of passengers, the network feature, and trip characteristics. The research results reveal the mechanism of route choice and give crucial information concerning operation and management. Among these data, in-vehicle time, transfer time and number of transfers are widely imported, including De et al., Zhang et al. and Eluru et al. [4–6]. Furthermore, a host of latent variables are also discussed to perfect the modeling. Hochmair [7–8] and Reveau et al. [9] emphasized the factors related to topology indicating the deviation between origins and destinations and made comparisons between the effects on route choice conducted by different types of schematic maps. These studies both demonstrated that the deviation has a negative effect on route choice and the schematic maps actually play a role in passengers’ finding the shortest route. Leurent, Tirachini et al., and Pursula et al. [10–12] studied the factors related to the perception of comfort, including load density and seat occupancy, showing that passengers would like to pursue room and seats to feel comfortable during trips.

As for the modeling of route choice behavior, the multinomial logit (MNL) model is the most widely applied for the analysis of route choice behavior because of its easy application. However, due to the assumption that the error component follows the identical and independent Gumbel distribution (IID), the biases exist in parameter estimations in most cases when facing the overlapping problem between alternatives in transportation networks [13]. As a consequence, a host of revised models were proposed to overcome the defect brought by the independence of irrelevant alternatives (IIA) property, mainly referring to overlapping problem in route choice, such as C-logit, path sized logit (PSL), and cross-nested logit (CNL), etc. [14–19]. It is worth noting that the overlapping issue also exists in the Metro network, which is easy to be neglected in route choice modeling [20]. Thus, it is essential to pay attention to the overlapping problem in the study of route choice behavior in networks to advance the accuracy.

Intuitively, it is viewed that the penalty effect of transfer time on route choice behavior is different under various number of transfers. Concerning transfer, different studies regard the transfer variables in various ways. Liu et al. [21] simply considered the transfer factor as transfer time and the number of transfers. Bovy and Hoogendoorn-Lanser [22] subdivided the transfer factor into walking time and waiting time, combining the dummy variable of trip chains. Raveau et al. [20] made an elaborate investigation on the transfer factor, including ascending transfers, even transfers, descending transfers, assisted transfers, semi-assisted transfers, and non-assisted transfers, which are significant in route choice behavior. However, these studies do not conjunctively consider transfer time and the number of transfers, so the difference of the penalty effect on the transfer time under various transfers has not been sufficiently investigated yet. Under the inspiration of the studied done by Bovy and Hoogendoorn-Lanser [22–23], which divided the service frequency into high frequency and low frequency and showed that the penalty effect of low service frequency is severer on passengers’ transfer choice, the concept of “perceived transfer threshold” is proposed in this paper intending to explore varying degrees of penalty in passengers’ utility brought by transfer time when passengers are facing different numbers of transfers.

Therefore, the objective of this study is to verify the rationality of the perceived transfer threshold for passengers and perfect the model formulation to improve the explanatory abilities and advance the understanding of passengers’ behaviors when choosing routes in a Metro network, as well as quantifying the impacts of the explanatory variables. Additionally, the paper aims to demonstrate how to apply the research achievement of route choice behavior analysis to practical engineering applications.

The rest of the paper is organized as follows. The “modeling” section states the specification of model including model structure and description of explanatory variables, particularly focusing on the construction of a utility function considering the perceived transfer threshold. The “data” section gives an introduction on the data specification including network information, route choice data, and a set of alternative routes. The “results and analysis” section provides the results and analysis of models applied to the Guangzhou Metro, in terms of the analysis in the perceived transfer threshold and calibration results with comparison against based models as reference. The “application example” shows a practical application case of route choice behavior analysis, using the research results to implement personalized route planning in the Metro network. The “conclusions” section presents the concluding comments to the study.

## Modeling

In this study, several factors are considered to explain the process of decisions made by Metro passengers among different characteristics of the alternative routes based on a PSL model structure to address the overlapping problem. Additionally, in the model, the stress is to form the structure that can describe penalty difference of transfer time under various transfers.

### Model structure

In existing studies, an MNL model presenting a simple mathematical structure and ease of estimation is widely applied to the analysis of passengers’ behavior due to the property of IIA, which holds that the ratio of the choice probabilities of any two alternatives is unaffected by the systematic utilities of any other alternatives. [24] However, on the contrary, one of the limitations existing in the MNL model brought by IIA property is that it does not consider any correlation between alternatives, which leads to misestimates of the probabilities. This correlation reflected in route choice is the overlap of alternative routes in an OD pair, and the misestimates become serious as the overlap becomes extended.

To address this issue, different modified models have been proposed. In this paper, due to the simple analytical formulation, the limited computational effort required for path size calculation and the easy estimation process, a PSL model is chosen as the functional form including a “path size” factor, which helps in capturing the correlation between alternatives and correcting the models’ fitness and predictions by lowering the probabilities of choosing similar alternatives in the MNL utility function. The functional form for the PSL is given in Eq (1), where $P_{k}^{rs}$ is the probability of choosing route *k* from *K*_*rs*_, a set of alternatives between OD pair *rs*, $V_{k}^{rs}$ represents the deterministic part of the utility function, generally assumed to be a linear function of the attributes as shown in Eq (2), where *β*_*i*_ represents the parameter of each attributes.

$$P_{k}^{rs}=\frac{\text{exp}\left(V_{k}^{rs}+\beta_{ps}\text{ln}PS_{k}\right)}{\sum_{j\in K_{rs}}\text{exp}\left(V_{j}^{rs}+\beta_{ps}\text{ln}PS_{j}\right)},k\in K_{rs}$$ (1)

$$V_{k}^{rs}=\sum_{i}\beta_{i}\cdot X_{i}$$ (2)

In addition, *β*_*ps*_ is the parameter of the path size factor, *PS*_*k*_, formulated by Ben-Akiva [25] as shown in Eq (3), where *L*_*k*_ is the length of route *k*, *l*_*α*_ is the length of link *a*, *τ*_*k*_ is the set of links in route *k*, *δ*_*αj*_ is the link-path incidence dummy, i.e., one if path *j* uses link *a*, and zero otherwise.

$$PS_{k}=\sum_{\alpha \in \tau_{k}}\frac{l_{\alpha }}{L_{k}}\cdot \frac{1}{\sum_{j\in K_{rs}}\delta_{\alpha j}}$$ (3)

Thus, all values of parameters in utility function (*β*_*i*_ and *β*_*ps*_) can be calibrated using the maximum likelihood method.

### Model specification

As mentioned in the introduction, many significant factors are considered to manifest the effect on passengers’ route choice behavior based on the theory of discrete choice model. This paper focuses on investigating passengers’ route choice preferences considering in-vehicle time, transfer time, number of transfer, angular cost, and average congestion, as well as age and network knowledge. Meanwhile, compared with previous work, the existence and rationality of the perceived transfer threshold is emphatically analyzed in order to describe the varying degrees of penalty in Metro passengers’ utility brought by transfer time when passengers face different numbers of transfers. Thus, the definitions of all the explanatory variables involved in the model and how the utility function with the perceived transfer threshold is built are stated in this segment.

### Explanatory variables

The time factor is one of the most significant and traditional variables in explaining the preference of passengers’ route choice behavior. Empirically, passengers tend to take the route with shortest time among alternatives. In this paper, time related factors include in-vehicle time and transfer time to address the different effects of the two components on passengers’ route choice, respectively.

In addition to the transfer time, the number of transfers is also considered to describe the displeasure brought by the total number of transfers of each alternative. This route character is associated with both psychological and physiological perceptions of passengers, resulting in the fact that passengers tend to take the route with the fewest number of transfers to avoid fatigue during the trip.

Angular cost is also taken into account, which is proposed to measure the deviation of a specific route away from the line linking the origin and the destination, in order to reflect the preference that passengers tend to choose the most direct routes from origin to destination. This variable was first proposed by Reveau et al. [9] and improved by Zhang et al. [5]. Zhang’s formulation of angular cost is adopted for the reason that the unit penalty effect of angular cost will arise along with the increase of route deviation, which is verified to be more consistent with the actual situation. The formulation is given by Eq (4), where ${\text{AC}}_{k}^{rs}$ represents the angular cost of route *k* between origin *r* and destination *s*, *l*_*i*_ is the length of link *i*, *m* is the number of links in route *k*, and *α*_*i*_ is the angle formed between the destination station, the first station of link *i* and the last station of link *i*.

$${\text{AC}}_{k}^{rs}=\sum_{i=1}^{m}l_{i}*\text{tan}(\alpha_{i}/4)$$ (4)

Average congestion is included in the model to investigate how the perception of crowding experienced by passengers during trips influences their route choice decisions. Particularly, this variable is defined in the form of time-weighted average occupancy ratio of compartment as shown by Eq (5),

$$Q_{k}^{rs}=\frac{\sum_{i}\theta_{i}\cdot T_{i}}{\sum_{i}T_{i}}$$ (5)

where $Q_{k}^{rs}$ represents the average congestion, *T*_*i*_ represents travel time in section *i* (including in-vehicle time and stoppage time), *θ*_*i*_ represents occupancy ratio of section *i*.

Network knowledge is a dummy variable set on the utility function(s) with corresponding route(s) that possess(es) the least travel time and measured by travel frequency (1 if travel frequency is no less than 8 times per week, 0 otherwise) to investigate how the familiarity for the network influence the route choice behavior. The frequency threshold “8 times” is determined by the passengers’ average travel frequency calculated from the survey sample. It is assumed based on a common sense that passengers who frequently take metro are familiar with the network and level of service so that they can readily choose the routes with least time.

Age variable is set on the utility function(s) whose corresponding route(s) possess(es) the least number of transfers in order to capture the peculiar behavior formed by passengers with different age. In the paper, age is also a dummy variable. It is assumed that elder passengers tend to take the routes with fewer transfers. Therefore, it equals 1 when the age of passenger is more than 50, and 0 otherwise.

Beyond the variables above, due to the limitation of the survey as well as the emphasis on the exploration of perceived transfer threshold, other latent attributes are not included. In addition, the fare factor is not incorporated since all alternative routes between a given OD pair share the same monetary cost. The cost is determined only by origin and destination stations.

### Perceived transfer threshold

**Fig 1:**
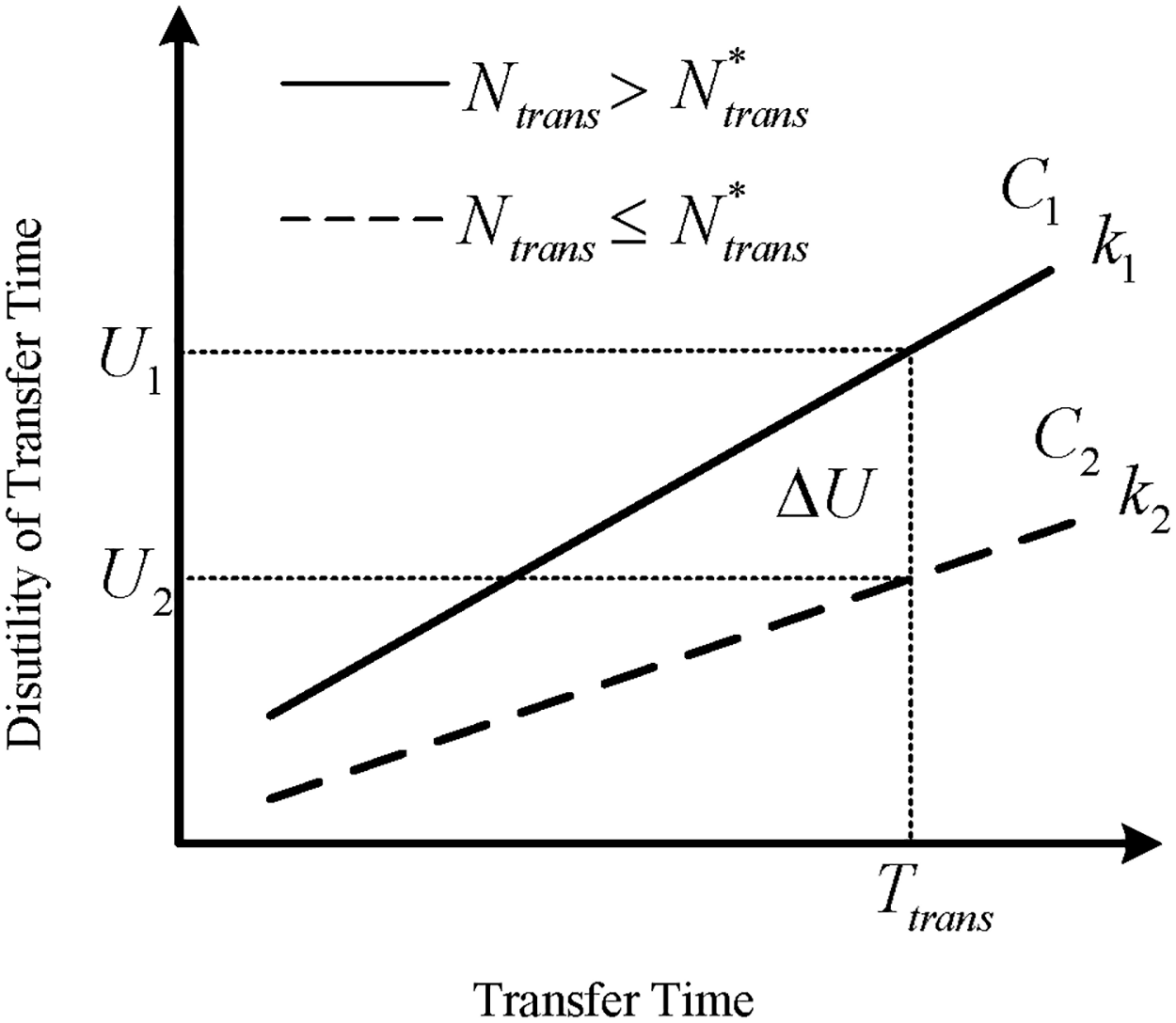
Diagram of transfer time disutility considering perceived transfer threshold.

As mentioned, Metro passengers’ perception of transfer is related to transfer time and the number of transfers, both of which have a negative influence on route choice decision making, whereas both factors have rarely been integrated in considering. In this paper, it is assumed that the disutility function of transfer time is segmented by the specific number of transfers, “perceived transfer threshold, $N_{trans}^{*}$”, as the critical point perceived by passengers in route choice decision, which purports that passengers perceive an excess penalty of transfer time when facing high number of transfer, as depicted in Fig 1, where x-coordinate represents transfer time, y-coordinate represents the perception disutility of transfer time, and the solid curve (*C*_1_ when $N_{trans}>N_{trans}^{*}$) and imaginary curve (*C*_2_ when $N_{trans}\leq N_{trans}^{*}$) show the disutility difference respectively under different number of transfers.

It is obvious that there is a disutility different (Δ*U*) between *C*_1_ and *C*_2_ (corresponding to *U*_*1*_ and *U*_*2*_) when experiencing the same *T*_*trans*_ but different *N*_*trans*_ for individuals. This diagram shows that passengers feel more negative when experiencing a high number of transfers, which will lead to a high slope rate (as *k*_1_ and *k*_2_ shown in the diagram) in passengers’ disutility function of transfer time, resulting in the probability decrease of the route to be chosen. Due to the adoption of a linear function of the attributes in utility function, *k*_1_ and *k*_2_ are fixed values in this paper.

Thus, to verify the assumption and investigate the different penalty effect brought by transfer time on Metro passengers’ route choice behavior thoroughly, on the basis of considering passengers’ different perception on number of transfers, the utility function is proposed as shown in Eq (6),

$$\begin{array}{ll} V_{k}^{rs} & =\beta_{1}\cdot T_{iv}+\beta_{2}\cdot N_{trans}+\beta_{3}\cdot \left(N_{trans}\leq N_{trans}^{*}\right)\cdot T_{trans}+\beta_{4}\cdot \left(N_{trans}>N_{trans}^{*}\right)\cdot T_{trans}\\ & +\beta_{5}\cdot AC_{k}^{\text{rs}}+\beta_{6}\cdot Q_{k}^{rs}\text{+}\beta_{7}\cdot Age+\beta_{8}\cdot NK \end{array}$$ (6)

where *β*_1_~*β*_8_ are the parameters for each explanatory variables, *T*_*iv*_ represents in-vehicle time, *N*_*trans*_ represents number of transfers, *T*_*trans*_ represents transfer time, $N_{trans}^{*}$ represents the value of perceived transfer threshold, *Age* represents age dummy variable (1 if older than 50 years old, and 0 otherwise), *NK* represents the level of network knowledge. The logical judgment to the perceived transfer threshold adopts Boolean variables, i.e., $N_{trans}\leq N_{trans}^{*}$ equals one when number of transfer is less than the perceived transfer threshold, and zero otherwise.

## Data

### Network and operation information

Based on the survey of the Guangzhou Metro network and operation conducted in 2013, the Metro network of Guangzhou city includes 8 lines in operation, with 123 stations, 12 transfer stations and 15,006 OD pairs altogether under a complete network condition, which brings the complexity and multiplicity to the behavior pattern of passengers’ route choice. The coordinates of each station, distances of each link, and all the transfer time at transfer stations are obtained. Note that the transfer time at transfer stations is specifically the average transfer time and measured by Guangzhou metro stuff.

**Fig 2:**
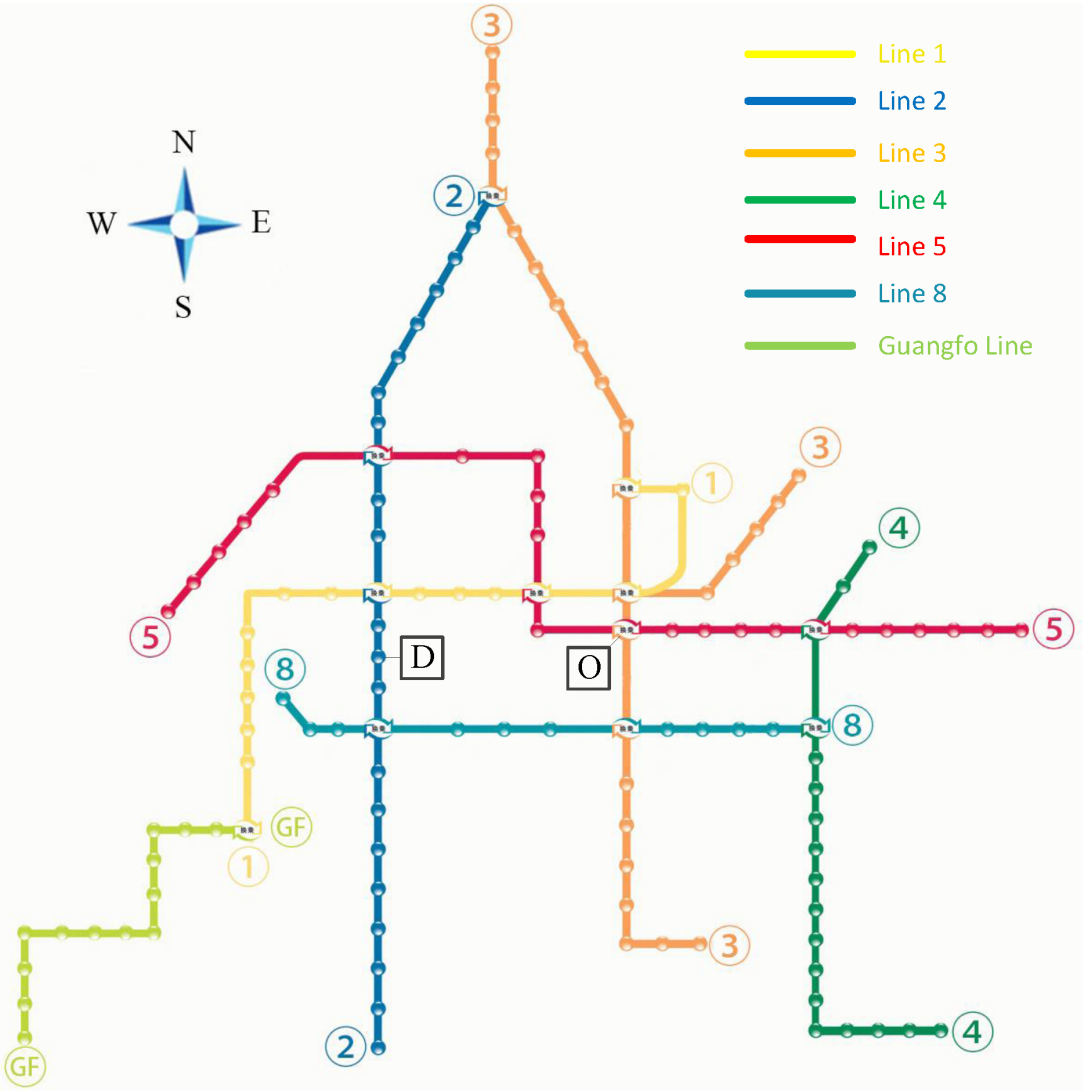
Diagram of topology in Guangzhou metro (July 2013).

The Metro operation survey involves the operation schedule and the passenger flow data provided by Guangzhou metro. The passenger flow data that is originally collected by automatic fare collection (AFC) system includes one month’s section passenger volume. By these categories of data, we can calculate the in-vehicle time, transfer time, number of transfers, angular cost, and average congestion of each route in any OD pairs, which can be used in generating set of alternatives. The topological structure of Guangzhou Metro in 2013 is depicted in Fig 2.

### Revealed preference (RP) survey data

The investigation was designed and implemented by Beijing Jiaotong University and assisted by Guangzhou metro staff. The RP data of passengers’ route choice was collected in the form of questionnaire at each transfer station in 2013, when there was no new line access in the Metro network. In the questionnaire, we asked and recorded passengers about the origin, destination, and all the transfer stations in sequence of each individual trip as well as travel frequency, travel purpose and the socio-economic attributes (such as age, occupation, gender, income), and finally data for 14,572 valid individual trips were obtained.

The personal attributes statistical results are listed in Table 1.

**Table 1:** Personal attributes statistical results.

Personal Attributes	Statistical Results
Gender	Male: 53.1%; Female: 46.9%
Age	Less than 18 years old: 11.8%; 18–25 years old: 45.7%; 25–35 years old: 32.8%; 35–50 years old: 7.6%; More than 50 years old: 2.1%
Occupation	Government: 26.21%; Enterprise and public institution:46.89%; Student: 12.62%; Retiree: 1.90%; Freelance: 7.20%; others: 5.18%
Income	Less than 1500 Yuan: 20.4%; 1501–3000 Yuan: 28.0%; 3001–5000 Yuan: 29.5%; 5001–10000: 17.5%; More than 10000: 4.7%

It can be seen that the distributions of all personal attributes are quite reasonable. Thus, the sample provides a basically adequate coverage of the travel population, and the mode preference observations can be used in model estimation to quantify the impact of different attributes.

### Set of alternative routes

The generation of alternative routes set is the basis of route choice behavior analysis. Because the modeling data used is RP data, it means that by the investigation we only have origin stations, destination stations, and transfer station(s) in a trip, which together determine unique routes as choice results. Different from SP data, we do not have the clear alternative set of routes, but the advantage of the RP data is that the choice results are revealed in behavior. Thus, a database of feasible alternatives is required.

Moreover, the validity of alternative set is essential for improving the precision of the model. For constructing valid set of alternative routes, many approaches have been developed. Among them, the double-sweep algorithm is employed in the paper due to the advantage of satisfactory time and space complexity, which screens out *k*-shortest paths between origins and destinations with constraints of passengers’ tolerances towards travel time [26–27].

Thus, applying double-sweep algorithm, the alternative routes set of the Guangzhou Metro is generated with a minimum number of one and up to six different routes in each of the 15,006 OD pairs, summarized in Table 2 along with the distribution of observations owning multiple alternative routes, and an example of the data structure for alternative route set is shown in Table 3.

**Table 2:** Statistics of the distribution of alternative routes in OD pairs.

Alternative Routes	Existing OD Pairs (Ratio)	Observations (Ratio)
1	2,939 (19.6%)	0 (0.0%)
2	2,478 (16.5%)	5,145 (35.3%)
3	876 (5.8%)	1,039 (7.1%)
4	989 (6.6%)	1,402 (9.6%)
5	2,021 (13.5%)	2,240 (15.4%)
6	5,703 (38.0%)	4,746 (32.6%)
Total	15,006 (100.0%)	14,572 (100.0%)

**Table 3:** Statistics of the distribution of alternative routes in OD pairs.

Origin ID	Destination ID	Route No.	Transfer station ID	In-vehicle time	Transfer time	Number of transfer	Angular cost	Route distance	Average congestion	PS factor
…	…	…	…	…	…	…	…	…	…	…
1	15	1	-	1,863	0	0	72.16377	16,100	0.389773	0.210606
1	15	2	75-94-	2,067	510	2	83.56652	19,305	0.410016	0.271031
1	15	3	12-55-21-	1,979	690	3	94.81082	17,783	0.430009	0.239344
1	15	4	4-36-16-	2,271	780	3	119.7047	20,730	0.390502	0.556126
1	15	5	12-55-78-94-	2,228	990	4	106.2136	20,988	0.43642	0.290535
1	15	6	3-26-46-21-	2,417	870	4	163.0663	22,969	0.411183	0.603574
…	…	…	…	…	…	…	…	…	…	…

## Results and analysis

Combining the choice data from RP survey and the sets of alternative routes, 14,572 sets of route choice modeling data were obtained. Specifically, 13,572 sets of choice data were used for calibrating model parameters, and another 1,000 sets of data serve for the hit ratio in this section. The evaluation of the proper perceived transfer threshold is also emphatically discussed to better grasp the feature of passengers’ route choice behavior.

Two based models, M1 and M2 (M1 is built in the form of an MNL model, and M2 is a PSL model without the consideration of the perceived transfer threshold) are estimated simultaneously to make comparisons in this section. A multicollinearity test is conducted to detect the multicollinearity between key variables. The Tolerance (TOL) and Variance Inflation Factor (VIF) are used as the criteria. Empirically if the value of TOL is greater than 0.1 or VIF is less than 10, it can be viewed as the multicollinearity is acceptable [28–31]. The test results are shown in Table 4. According to the test result, the VIF of the variables are almost within 5, and the TOL of them is around 0.2. In other words, there is an acceptable collinear relationship between the variables.

**Table 4:** Multicollinearity statistics of model variables.

Variable	Multicollinearity statistics	
	Tolerance	VIF
*T*_*iv*_ (min)	0.265	3.776
*N*_*trans*_	0.211	4.735
*T*_*trans*_ (min)	0.198	5.042
*AC* (km)	0.350	2.855
*Q*	0.577	1.732
*PS*_*k*_	0.515	1.942

Table 5 shows the comparison of parameter estimation results of these two models. The estimated parameters are all with reasonable significance and expected signs in both models. Note that M2 shows a better performance in log-likelihood and adjusted *ρ*^2^. In addition, the estimation results also show that the path size factor (with a positive factor, 0.5815) has a positive effect on route choice. In other words, the utility of route choice decreases when the overlapping issue becomes severer. This sign indicates that the PSL model structure with the consideration of path size factor that is capable of addressing the overlapping issues has better performance in describing passengers’ route choice behavior rather than the MNL model structure. Therefore, the PSL model structure is appropriate for modeling the route choice behavior in the Metro network apart from the road transportation network.

**Table 5:** Estimation results for the MNL and PSL models of passengers’ route choice (t-values in parentheses).

Variables	M1	M2
*T*_*iv*_ (min)	-0.2466 (-21.702)	-0.2679 (-21.989)
*N*_*trans*_	-1.7070 (-22.933)	-1.8669 (-23.078)
*T*_*trans*_ (min)	-0.4391 (-17.834)	-0.4257 (-17.185)
*AC* (km)	-0.6953 (-4.085)	-0.5414 (-3.126)
*Q*	-2.9468 (-9.366)	-1.4945 (-3.562)
*Age*	0.9701 (1.762)	1.0031 (1.791)
*NK*	0.3761 (7.923)	0.3708 (7.798)
*PS*_*k*_	—	0.5815 (5.264)
Sample size	13572	13572
Log-likelihood	-4751.56	-4597.73
Adjusted *ρ*^2^	0.7004	0.7101

Note: when absolute t-value is greater than 1.96 (1.65), the significance level is at 5% (10%).

To make a thorough investigation on the assumption that passengers perceive different disutility on transfer time under different number of transfers and to analyze the process of decision making on route choice more precisely, the perceived transfer threshold is taken into account to describe the different penalty effect on route choice perception utility. What is needed to be done is not only the certification on the existence of the perceived transfer threshold but also to obtain a proper number of transfers as the perceived transfer threshold to improve the accuracy and explanatory ability of model as well. Therefore, four PSL models with different perceived transfer thresholds are estimated respectively to discover the proper perceived transfer threshold on describing the behavior of passengers’ route choice, as M3 to M6 shown in Table 6, where *T*_*trans*1_ represents the parameter of transfer time with a lower number of transfers, and *T*_*trans*2_ represents the parameter of transfer time with a higher number of transfers.

**Table 6:** Estimation results for the PSL models under different perceived transfer thresholds (t-values in parentheses).

Variables	M3 ($N_{trans}^{*}=1$)	M4 ($N_{trans}^{*}=2$)	M5 ($N_{trans}^{*}=3$)	M6 ($N_{trans}^{*}=4$)
*T*_*iv*_ (min)	-0.2678 (-21.892)	-0.2675 (-21.960)	-0.2675 (-21.940)	-0.2679 (-21.983)
*N*_*trans*_	-1.3677 (-13.579)	-1.8224 (-21.252)	-1.8624 (-22.909)	-1.8670 (-23.078)
*T*_*trans*1_ (min)	-0.3030 (-10.396)	-0.4243 (-17.122)	-0.4250 (-17.144)	-0.4257 (-17.182)
*T*_*trans*2_ (min)	-0.4587 (-18.120)	-0.4381 (-16.770)	-0.4355 (-14.160)	**-0.6331 (-0.037)**
*AC* (km)	-0.6704 (-3.812)	-0.5299 (-3.061)	-0.5401 (-3.122)	-0.5411 (-3.126)
*Q*	-1.1953 (-2.829)	-1.5101 (-3.604)	-1.4959 (-3.567)	-1.4941 (-3.561)
*Age*	0.9835 (1.789)	1.0071 (1.793)	1.0027 (1.788)	1.0039 (1.792)
*NK*	0.3820 (7.965)	0.3735 (7.861)	0.3722 (7.819)	0.3708 (7.797)
*PS*_*k*_	0.6203 (5.563)	0.5793 (5.253)	0.5807 (5.259)	0.5816 (5.265)
Sample size	13,572	13,572	13,572	13,572
Log-likelihood	-4,324.94	-4,514.22	-4,515.26	-4,515.42
Adjusted *ρ*^2^	0.7273	0.7153	0.7153	0.7153

Note: when absolute t-value is greater than 1.96 (1.65), the significance level is at 5% (10%).

It is obvious that M3 to M5 have satisfying goodness of fit, and the parameter estimations are all with reasonable significance. By contrast with M3~M5, the estimation result of M6 is unacceptable for the reason that the variable *T*_*trans*2_ is obviously not significant (t-value, -0.037), in spite of a satisfactory adjusted goodness of fit. This finding implies that there are few passengers who would like to choose routes with more than four times of transfers, which reflects that passengers tend to choose convenient and fast routes with fewer transfers due to strict arrival time constraints.

Comparing M3~M5 with M2, although all the log-likelihoods of M3 to M5 are better than the one of M2, the accuracy of M4 and M5 (when the $N_{trans}^{*}$ equals 2 and 3) are not as significantly improved as M3 (0.7273 in M3 is superior to 0.7153 in other models). Namely, M3 has the best log-likelihood and the adjusted *ρ*^2^ when the perceived transfer threshold values 1, so that it is justifiable to take M3 as the final proposed model. Moreover, although all variables of the three models have significance in the estimation, bias can still exist in the results, which indicates the importance in obtaining an appropriate value of perceived transfer threshold, as well as the improvement in the model accuracy.

To further examine the accuracy, hit ratios of the proposed model (M3) and two base models (M1 and M2) are calculated to be 88%, 83.5% and 85.5% respectively, using another 1,000 samples of route choose data. The hit ratio is a statistical index for testing the consistency of observations and predictions in route choice. By comparing the results of hit ratios, it can be easily concluded that the proposed model has better accuracy than the other base models. This result verifies the model with consideration of perceived transfer threshold is more in line with reality.

Based on the estimation result from the final proposed model (M3), the result for age variable (1 if older than 50 years old, otherwise 0) is of concern, whose effect is positive. It signifies age variable has a positive effect on the utility when passengers choose the routes with the least number of transfers. When age variable equals 1, the routes with the least number of transfers comparing with other routes will have an extra increase in the utility. This indicates that elder passengers prefer routes with as few transfer stations as possible. In addition, with regard to the result of network knowledge measured by the passengers’ travel frequency, it has a positive effect on route choice process, which reflects that passengers who take Metro regularly find it easier to be familiar with the information of the network, particularly on finding the shortest route. Obviously, the age and network knowledge of passengers actually influence the behavior of route choice. Understanding the effect of these personal attributes can be helpful in providing different passengers with personalized services, such as the personalized route planning.

Concerning the result of angular cost and average congestion variables, intending to describe the degree of comfort experienced by passengers during trips, they both have the right sign of a negative effect, which manifests that passengers are inclined to take a direct route or uncrowded route so that they can alleviate the negative feelings in both physiological and psychological dimensions brought by deviation and congestion of route.

To elucidate the route choice behavior, it is important to quantify the impact of the attributes to figure out the weight of different attributes considered during route choice process. Usually, the monetary valuations are calculated to analyze the weight difference. However, the monetary valuations for various variables are infeasible to acquire due to the same fares of the alternatives in the same OD pairs. Thus, the equivalent in-vehicle time coefficients (*EITC*) between variables are derived instead according to the Eq (7) to analyze the attitudes of passengers towards various variables visually, where *β*_1_ represents the parameter of in-vehicle time and *β*_*i*_ represents the parameter of variable *i*.

$$EITC_{i}=\beta_{i}/\beta_{1}$$ (7)

The equivalent in-vehicle time coefficients are presented in Table 7. It is not difficult to determine that passengers care the most about the number of transfers and average congestion, equivalently 5.11 and 4.46 minutes of in-vehicle time, respectively. This phenomenon reveals that passengers during trips are more willing to choose routes with less transfers and density to avoid the physical exhaustion.

**Table 7:** Equivalent in-vehicle time coefficients.

Variables	Parameters	*EITC* (min)
*T*_*iv*_ (min)	-0.2678	1.00
*N*_*trans*_	-1.3677	5.11
*T*_*trans*1_ (min)	-0.3030	1.13
*T*_*trans*2_ (min)	-0.4587	1.71
*AC* (km)	-0.6704	2.51
*Q*	-1.1953	4.46

As for transfer time, it can be judged that the penalty of transfer time is greater than the in-vehicle time, which suggests that passengers are more sensitive to transfer time, so that passengers always tend to choose routes with less transfer time. Beyond that, by comparing the *EITC* of *T*_*trans*1_ (1.13 min) with *T*_*trans*2_ (1.71 min), it is significant that the transfer time with more transfers shows a more serious penalty effect than the *EITC* of transfer time with only a few times of transfers, and the penalty of transfer time with a high number of transfers is 1.5 times (1.5 = 1.71/1.13) of the one with a low number of transfers, which indicates that passengers feel more negative when experiencing a high number of transfers. This finding verifies the rationality of perceived transfer threshold that the judgment on perceived transfer threshold actually exists in the process of route choice decision making, and it can better describe the behavior of passengers.

## Application example

The results of route choice behavior analysis are the core to support operation management and advanced passenger information service [32], which can be applied to various aspects, such as passenger flow assignment, transfer volume forecasting, personalized route planning, and fare clearing, etc. As an application example of route choice behavior analysis in this study, a personalized route planning case is presented to demonstrate how the results of route choice behavior analysis play an instructive role in Metro operation management.

### Personalized route planning case

Conventional route planning strategies usually adopt the principle based on a specific single goal, such as the optimal time, the minimum transfer, or the least cost, which is not able to reflect the comprehensive influence of various factors on passengers’ route choice [33–36]. In contrast, the personalized route planning method is not only able to take various factors of LOS variables into consideration but also passengers’ personal attributes. Therefore, a personalized route planning case is presented in this paper to show the advantage of comprehensive consideration of various factors.

To implement the route planning, two ways can be applied. One is based on path search algorithms, such as Dijkstra algorithm, etc., which accumulate the generalized travel time of all the factors in each link and maintains the link information of the optimal routes. The other way is to calculate and compare the generalized travel time of each route in a specific OD pair based on an existing route set of alternatives and then choose the route with the optimal generalized travel time. With the intention of simplifying the calculation process, an alternative route set has been generated in advance with ten alternative routes in each OD pair. All the routes are searched according to travel time as the standard and ranked on the basis of travel time. Meanwhile, all the feasible routes to be searched must satisfy the restricting condition of the absolute travel time threshold and the relative travel time threshold, which requires the travel time of the routes to be searched not to exceed a specific travel time or specific times of the minimum travel time [37]. The principles above will basically guarantee all the feasible routes to be concluded in the alternative route set.

Then, based on the results of route choice behavior analysis, the generalized travel time function of Metro routes can be derived from the estimated utility function. All the parameters in the generalized travel time function can be obtained by calculating the *EITC* on the basis of model estimation. In this case, it is assumed that route selection strategies considering various factors can be addressed by passengers and the generalized travel time is calculated under a certain equation. Accordingly, on the basis of the feasible alternative route set, the generalized travel time of routes in any OD pairs can be calculated considering different personal attributes.

In the following, a given OD trip, departing from station O, “Zhujiangxincheng”, and arriving at station D, “Shiergong”, shown in Fig 2, is chosen as an example to implement the personalized route planning. Two imaginary passengers are assumed. Passenger A is assumed to be 55 years old and take Metro frequently (at least 10 times per week), and Passenger B is assumed to be 25 years old and to be not quite familiar with the Metro network (no more than twice per week). Applying the personalized route planning method, the information of the planned routes involved in this case are listed in Table 8, and the route planning results for both imaginary passengers are shown in Table 9.

**Table 8:** Planned routes information.

Route No.	Line Information	LOS Variables				Network Feature	
		*T*_*tra*_ (min)	*T*_*trans*_ (min)	*N*_*trans*_	*Q*	*AC* (km)	*PS*_*k*_
1	Line3-Line8-Line2	13.73	8	2	0.565	0.762	0.601
2	Line5-Line2	25.25	5	1	0.468	0.961	0.458
3	Line3-Line1-Line2	16.12	5.67	2	0.438	0.423	0.491

**Table 9:** Route planning results.

Scenario	Method	Description	Planning Result (Route No.)	Generalized Travel Time (min)
1	Minimum travel time	In general case	1	—
2	Generalized travel time without transfer threshold	For Passenger A: 55 years old, familiar	1	36.78
3	Generalized travel time with transfer threshold	For Passenger A: 55 years old, familiar	2	33.64
4	Generalized travel time with transfer threshold	For Passenger B: 25 years old, not familiar	3	35.03

**Fig 3:**
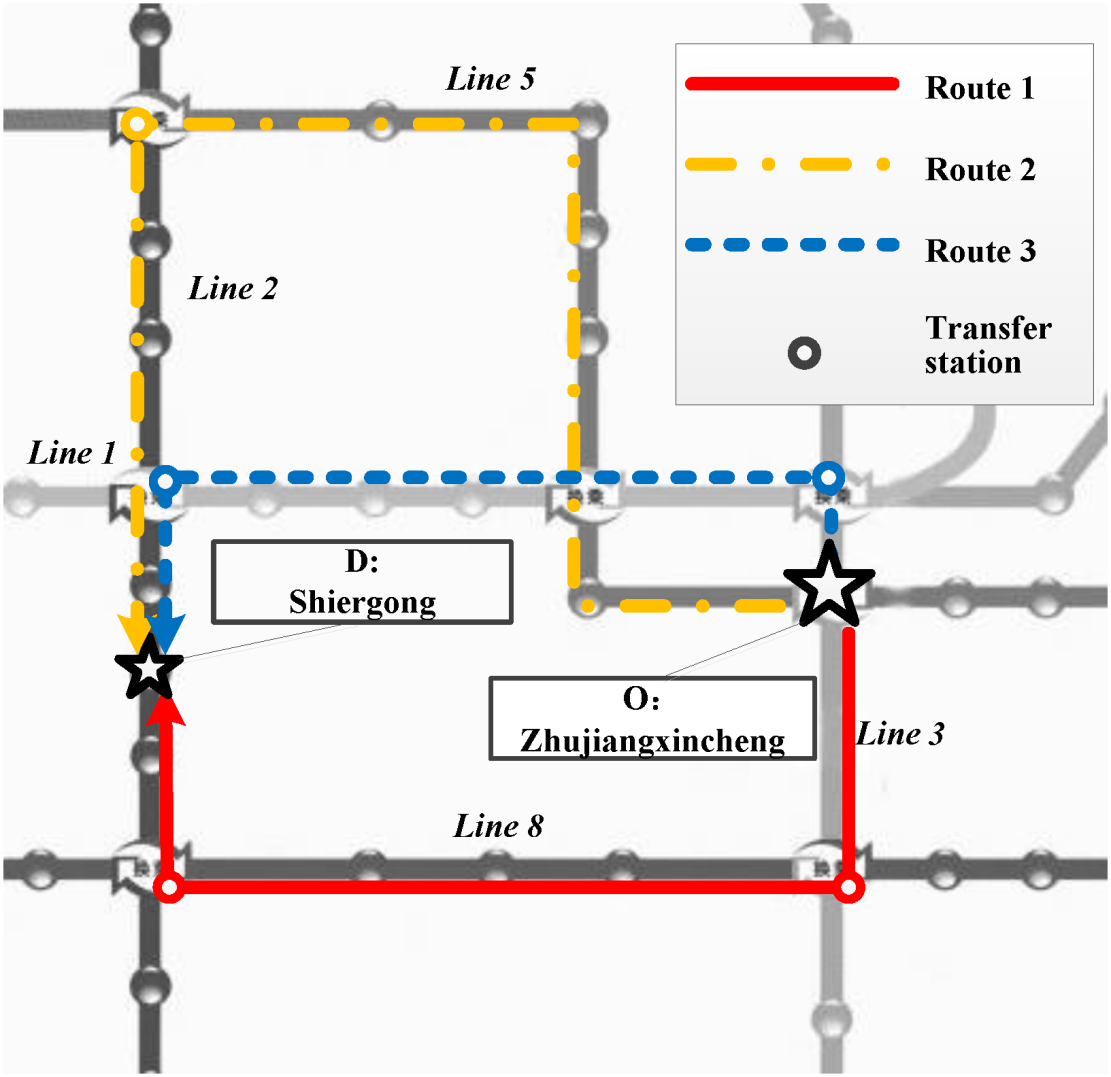
Schematic diagram of the planned routes.

To have a vivid image on the planned routes, which are numbered in sequence, the schematic diagrams are shown in Fig 3.

In general cases, the conventional route planning method gives a single planning result regardless of various scenarios. In contrast, it is obvious to see that the personalized route planning method provides differences in the planning results. Comparing the planning results of Scenario 3 with Scenario 2, the personalized route planning method considering the perceived transfer threshold gives the result of Route 2, probably because Passenger A with an older age trends to choose the route with a fewer number of transfers due to the higher penalty on utility when facing a higher numbers of transfers, and the weight of transfer is heavy for elderly passengers in the trade-off between the number of transfers and travel time. Thus, the personalized route planning method considering the perceived transfer threshold concerns adequately on passengers’ preference of transfer, and it may better meet the requirement of passengers according to a more comprehensive analysis on transfer.

In addition, based on the result comparison between Scenario 3 and Scenario 4, it is obvious that the personalized route planning method provides different results for different passengers. For such passengers as Passenger B who is young and not familiar with the network, they care less about the number of transfers and rely more on the display map to choose the trip route in the Metro network. As a result, the personalized route planning method provides Passenger B with the plan with less angular cost instead of the plan with minimum travel time in order to meet the route choice preference. In conclusion, it can be judged that the personalized route planning method is capable of disposing of the route planning task when confronted with various types of passengers and provide more reasonable route plan results.

## Conclusions

Route choice behavior analysis helps to elucidate how the factors affect passengers’ travel behavior, the research achievements of which have significant guidance in both operation management adjustment and passenger service promotion. This paper deeply investigates the passengers’ route choice behavior in the Metro system based on discrete choice theory and takes personalized route planning as an application case to show the practical engineering value of route choice behavior analysis, which is demonstrated to be capable of providing more reasonable planning results in different circumstances considering the route choice preferences. In the discussion of route choice behavior, this paper mainly emphasizes on the different penalty effect on transfer time perceived by passengers when facing different numbers of transfers. Meanwhile, the route-overlapping problem in the Metro network is also paid attention to, which is represented by the PSL model. Therefore, a route choice model is proposed in this paper, in which the utility function consists of level of service variables, socio-demographics, network knowledge, the path size factor denoting the penalty of route overlapping degree on the utility, and the logical judgment measuring the penalty effect on transfer time under different numbers of transfers.

Based on the RP survey data and network data collected from Guangzhou Metro, the proposed model is estimated, and the corresponding results are discussed. First, the estimations show that the route-overlapping problem significantly affects route choice behavior, and the PSL model shows a better performance in describing passengers’ route choice behavior than the MNL model in the Metro network. Second, different perceived transfer threshold values are assumed to find a proper value, and the influence of the perceived transfer threshold on the penalty effect on transfer time under different number of transfers is discussed. The estimation results show that the model with the perceived transfer threshold equal to 1 has the best performance in forecasting accuracy among the base model (without considering the penalty effect on transfer time) and the models with other perceived transfer threshold values. Therefore, with respect to route choice modeling in the Metro network, the penalty effect on transfer time under different numbers of transfers should be considered. Finally, the influences of other factors, such as age and network knowledge, on route choice preference are also discussed. The estimations show that passengers attach the most importance to the number of transfers and average congestion, which indicates that passengers tend to choose more comfortable routes to avoid fatigue brought by crowding and transfers. Meanwhile, the estimation result of angular cost shows a negative effect on route choice, which is consistent with findings by Raveau that the topological factor does influence route choice preference. Furthermore, it is interesting to find that the *EITC* of transfer time with a high number of transfers is 1.5 times of that with only a few times of transfers, which clearly reflects that passengers reveal more negative feelings on the transfer time with more transfers and would like to choose few times of transfers as a result.

## Supporting information

S1 File Revealed preference survey data. (for [rp-data.csv], Deletable).
(CSV)

S2 File Link information. (for [link data 2013.csv], Deletable).
(CSV)

S3 File Station information. (for [station data 2013.csv], Deletable).
(CSV)
